# Long-term analysis on the variance of extra-group paternities in rhesus macaques

**DOI:** 10.1007/s00265-017-2291-7

**Published:** 2017-03-17

**Authors:** Angelina V. Ruiz-Lambides, Brigitte M. Weiß, Lars Kulik, Colleen Stephens, Roger Mundry, Anja Widdig

**Affiliations:** 1 Junior Research Group of Primate Kin Selection, Department of Primatology, Max-Planck Institute for Evolutionary Anthropology, Deutscher Platz 6, 04103 Leipzig, Germany; 2 Behavioral Ecology Research Group, Institute of Biology, Faculty of Bioscience, Pharmacy and Psychology, University of Leipzig, Talstrasse 33, 04103 Leipzig, Germany; 3 Cayo Santiago Field Station, Caribbean Primate Research Center, University of Puerto Rico, P.O. Box 906, Punta Santiago, 00741 Puerto Rico; 4 Max-Planck Institute for Evolutionary Anthropology, Deutscher Platz 6, 04103 Leipzig, Germany

**Keywords:** Rhesus macaques, Extra-group paternity, Male group membership, Breeding group sex ratio, Female synchrony, Group instability

## Abstract

**Abstract:**

Extra-group paternity (EGP) has been described in various mammalian species; however, little is known about which factors contribute to the variation in EGP, as the majority of studies were restricted in time and the number of groups considered. Using longitudinal demographic and genetic data, we aim to investigate which factors predict rates of EGP in the free-ranging rhesus macaque population of Cayo Santiago, Puerto Rico (USA). Of the 1649 infants considered which were born into six social groups over 9 years, we identified an average of 16% of infants resulting from EGPs. We tested the influence of group size, breeding group sex ratio, female reproductive synchrony, and group instability on the occurrence of EGPs. Our results suggest a tendency for EGPs to increase as the proportion of females increased in larger groups, but no such effect in smaller groups. Furthermore, as group instability and female reproductive synchrony decreased, the number of EGPs tended to increase. Our results support the hypothesis that group structure affects the occurrence of EGPs, which might be mediated by male mating opportunities, male monopolization potential, and/or female choice.

**Significance statement:**

In several species, both sexes seek alternative reproductive strategies to enhance their reproductive success. For instance, females may pursue EGPs to potentially increase genetic compatibility with males, or males may seek EGPs to improve their mating opportunities. Our longitudinal analysis, including demographic and genetic data over 9 years of six social groups of rhesus macaques, revealed high variation in the occurrence of EGPs across groups and years, and this variation tended to depend on group characteristics such as breeding group size, sex ratio, female synchrony, and group instability. The data suggest that group structure affects the number of EGPs in this group-living primate. Our results show that EGPs can affect the distribution of paternity within social groups and should be taken into account when assessing reproductive success.

**Electronic supplementary material:**

The online version of this article (doi:10.1007/s00265-017-2291-7) contains supplementary material, which is available to authorized users.

## Introduction

The proportion of offspring fathered by males outside of the breeding group (i.e., extra-group paternity, EGP) or pair (i.e., extra-pair paternity, EPP) plays a critical role in increasing genetic variability of populations while shaping the evolution of complex social systems (Isvaran and Clutton-Brock 2007). In pair-living birds, EPP is a common phenomenon (Griffith et al. 2002); however, studies on group-living mammals show that EGP is also widespread among them. According to a review of 26 mammalian species, frequencies of EGP greater than 10% were found in 58% of the species investigated, regardless of their mating system (Isvaran and Clutton-Brock 2007). The extent of EGP varied from zero in mammalian species such as the California mouse, *Peromyscus californicus*, but rose to 60% in species such as the southern elephant seal, *Mirounga leonina*, and up to 80% in the red fox, *Vulpes vulpes*. Studies on multimale primate groups reported 7 to 81% of EGP across species, e.g., 7% in the chimpanzee, *Pan troglodytes* (Vigilant et al. 2001), 11% in toque macaques, *Macaca sinica* (Keane et al. 1997), 33% (Soltis et al. 2001) and 61% (Inoue and Takenaka 2008) in Japanese macaques, *M. fuscata*, 36% (Berard 1999) and 24% (Widdig et al. 2004) in rhesus macaques, *M. mulatta*, 42% in Verreaux’s sifaka, *Propithecus verreauxi verreauxi* (Lawler 2007), or also the complete absence of EGP, as in the yellow baboon, *Papio cynocephalus* (Alberts et al. 2006).

EGP is thought to enhance reproductive success (Lindstedt et al. 2007; South et al. 2007; Bonadonna et al. 2013) by increasing genetic heterogeneity and alleviating the negative fitness consequences of homozygosity (Strum 1982). In general, EGP seems more likely when males are unable to fully monopolize receptive females (van Noordwijk and van Schaik 2004), suggesting that females, under such scenarios, are able to seek surreptitious matings that could ultimately result in EGPs. Consequently, the rates of EGP within social groups should depend on the reproductive strategies that the individuals of a given group pursue. Previous studies detected several group parameters that predicted the extent of EGP (Heckel et al. 1999; Griffith et al. 2002; Cohas et al. 2006). For example, a relationship between the proportion of females in a social group and the number of EGPs was found in a study on Verreaux’s sifaka, where the number of offspring sired by resident males in a social group decreased when the sex ratio was biased toward females (Lawler et al. 2003). Similarly, it has been suggested that the sex ratio in troops of Japanese macaques influences mating success of both troop and nontroop males, as nontroop males increased their chances of mating when troop males were not able to successfully monopolize estrous females (Takahashi 2001). Across 26 mammal species, however, EGP was only weakly related to the sex ratio of breeders (Isvaran and Clutton-Brock 2007). Meanwhile, a review of 13 nonhuman primate populations found no significant association between the proportion of EGP and group size (van Noordwijk and van Schaik 2004). As a result, the relationship between the breeding group sex ratio, group size, and extra-group paternity is far from being resolved.

Furthermore, other factors have been suggested to influence rates of EGP. Across mammals, Isvaran and Clutton-Brock (2007) describe an increase in EGP occurrence with a shorter mating season and, consequently, increasing female synchrony. Similarly, van Noordwijk and van Schaik (2004) found a significant correlation between the degree of mating seasonality and the percentage of EGPs across nonhuman primates. Moreover, group cohesiveness and stability may impact the extent of EGP (Isvaran and Clutton-Brock 2007). The flux of males into and out of a group during a particular mating season may affect the cohesiveness of a group and is usually accompanied by increasing levels of aggressiveness (Strum 1982). Group instability through the loss of resident males due to deaths and emigrations, the addition of males through immigration, as well as the instability caused by intragroup aggression may, thus, be associated with variation in social dynamics and mating opportunities (Isvaran and Clutton-Brock 2007; Clutton-Brock and Huchard 2013). In particular, instability of the male hierarchy is likely to influence social dynamics by disturbances caused through direct competition over rank, in which males migrating into the new group may challenge the “top males” (van Noordwijk and van Schaik 2004; Marty et al. 2015). Furthermore, when the degree of female reproductive synchrony is high, other males than the alpha male should be able to get access to fertile females (Dubuc et al. 2011).

Finally, it should be taken into account that EGPs may be facilitated by characteristics of neighboring groups. In a wild population of Verreaux’s sifakas, with high density and high degree of overlap in group home ranges, extra-group fertilizations contributed significantly to the variation of male fitness (Lawler 2007). Similarly, in European badgers, *Meles meles*, greater presence of males in neighboring groups increased the likelihood of extra-group fertilizations, possibly facilitated by the high degree of intergroup movements (Annavi et al. 2014).

Although there is evidence that group structure affects the occurrence of EGP across mammals, systematic studies that assess sources of intraspecific variation (Isvaran and Clutton-Brock 2007) in rates of EGP are scarce. Almost all studies that have investigated EGP in mammals were either based on mating or paternity data in only one reproductive season or one group (but see Lawler 2007) and therefore cannot be used to address the sources of variation in EGP between groups or seasons. Importantly, short-term studies do not necessarily reveal the same effects as long-terms studies, which might, in fact, lead to contrasting results (Lindburg 1971; Alberts et al. 2003). In order to understand within-species variation in EGPs, it is important to account for variance over years and groups arising from, e.g., variation in social or ecological factors in time and space. It is crucial to cover multiple groups and several seasons in populations with detailed demographic, paternity, and group membership data.

Given the aforementioned limitations, our study attempts to improve our understanding of the relationship between EGPs and group characteristics in primates by analyzing 9 years of demographic and genetic data from a population of rhesus macaques comprising of six naturally formed groups. Rhesus macaques live in multimale-multifemale groups, with both males and females mating promiscuously during the mating season (Lindburg 1971). Females are philopatric and form stable matrilineal hierarchies (Gouzoules and Gouzoules 1987), while males disperse during adolescence to join other social groups (Sade 1972). Given that male rhesus macaques do not necessarily acquire their rank by direct contest but attain dominance frequently by means of a queuing system, alpha males seem neither the most attractive mates nor do they attain the highest paternity success in the group (Dubuc et al. 2011). High ranking males utilize mate-guarding as a tactic to monopolize females, yet this strategy results in only one third of fertilizations (Dubuc et al. 2012). The limitation of male monopolization potential suggests the opportunity for female mate choice (Dubuc et al. 2011) by searching for mates outside the social group, which might explain the considerable degree of EGP (on average 24%) observed in a previous study that included 6 years of data on a single social group of rhesus macaques (Widdig et al. 2004).

Our main goal was to investigate group-level variables that potentially influence the extent of EGP across groups and seasons. We analyzed EGP in a rhesus macaque population that has been systematically observed since 1956 and genotyped continuously since 1992. The chosen population offered an exceptional opportunity for analyzing the influence of the breeding group sex ratio, female reproductive synchrony, group size, and group instability on the occurrence of EGP by the availability of comprehensive longitudinal observational data, extensive paternity data, and strict criteria for group membership designation to determine rates of EGP. Based on the limitations of males to monopolize fertile females, in particular when females are clustered in greater numbers, we expected the proportion of EGPs to be larger with an increasing group size and proportion of females within a social group. Similarly, we predicted that, as the synchrony of female fertility increases, the number of EGP increases due to the challenge males encounter to successfully monopolize females during their fertile phase. Finally, we predicted that levels of EGP increase as groups become more unstable due to males being more engaged in male-male interactions and less in mate-guarding when group composition and structure change.

## Methods

### Study site

Data were collected at Cayo Santiago, a 15.2 ha island, located 1 km off the southeastern coast of Puerto Rico (18°09′ N, 65° 44′ W), which is inhabited by a population of rhesus macaques. All monkeys that were part of this study were direct descendants of the 409 founder rhesus macaques captured from 12 districts across northern India and released on the island in 1938 (Rawlins and Kessler 1986). Although no animals have been added to the population except through birth, no increase in inbreeding over time was found in a recent study using extensive genetic data (Widdig et al. 2017). In over 99% of births, females have a single offspring (Bercovitch et al. 2002), and interbirth intervals are approximately 12 to 24 months (Fooden 2000). During the study period (2004–2012), the population size (measured 1 day before onset of the annual birth season) was 913 ± 77 animals (mean ± SD, range 800–1016), and the population comprised six different, naturally formed groups that ranged in size between 57 and 284 at any point in time. Animals on Cayo Santiago are provisioned with high-protein commercial monkey chow of approximately 0.23 kg per monkey per day. However, 50% of their feeding time is spent on natural vegetation (Marriott et al. 1989). Water is provided ad libitum through automatic drinkers found around the island.

Daily census reports on detailed individual life histories have been recorded continuously since 1956. All records are stored in a demographic database which contains data on age, sex, maternal genealogy, birth, death, and group transfer for each individual. Data on all live births, deaths, and group transfers are reported immediately or within 2 days after the actual event occurrence (details below). Reproduction in rhesus macaques is seasonal and coincides with patterns of rainfall and variation in vegetation; therefore, births can be assigned to distinct birth cohorts (Vandenbergh and Vessey 1968; Hoffman et al. 2008).

Direct human contact with the monkeys is limited to the annual trapping season. During this period, tetanus primary immunizations are given to all yearlings and tetanus boosters are administered to all 2-year-olds (Kessler et al. 2015). Biological samples such as blood for paternity analysis are collected, and yearlings are tattooed for identification purposes. Moreover, to control for population size, some animals are removed from the island with different culling strategies used across years (Hernández-Pacheco et al. 2013). During the study period, approximately 83% of individuals removed were younger than 3 years of age; however, nearly all individuals were sampled for parentage assignment prior to removal.

### Parentage assignment

The genetic database for the Cayo Santiago population contains data for the majority of individuals born on the island since 1992 as well as many of the older animals. Parentage data were obtained by a combination of efforts between the Caribbean Primate Research Center (CPRC) and the University of Leipzig (UL) (details in Widdig et al. 2017). In brief, nearly the entire population has been sampled for genetic information using blood samples, although in recent years tissue, hair and fecal samples have also served as a source for genetic material of dead or alive individuals. To date, genetic information is available for 4641 animals, genotyped on up to 43 microsatellite markers (mean ± SD = 27.6 ± 1.6) (details in Widdig et al. 2017). Maternity, as derived from behavioral observations, could be confirmed genetically for 3946 of 3996 (98.7%) mother-offspring pairs, and genetically confirmed maternity was successively used in the paternity analyses.

A total of 2079 live births were recorded in the population during our study period (birth cohorts 2004–2012), from which 1733 (83.36%) individuals were genotyped. Individuals with no genetic material available (0.05%), individuals that either died within the first year of their life (14.62%) or who were removed together with their mother before reaching their first year of age (1.97%) were not included in the sample. Given that infant mortality is highest within the first year of life (Blomquist 2013), genetic sampling at 1 year of age in general was never complete for an entire birth cohort. In addition, primate mothers continue carrying dead infants for several days (e.g., Sugiyama et al. 2009); hence, sampling dead infants is extremely challenging under free-ranging conditions. Finally, we excluded 84 further infants from groups not present during the entire study period, either because the group was removed from the island or split, i.e., we used 1649 infant in the analysis.

We included all genotyped males in the paternity analyses that were older than 1250 days of age (based on the earliest age of male reproduction: Bercovitch et al. 2003) and that were present on the island when a given infant was conceived, i.e., at least 200 days before the day of birth of the respective infant (given a gestation length of 166.5 ± 7.4 (mean days ± SD) according to Silk et al. 1993). Based on these criteria, we identified a total of 388 potential sires for individuals born between 2004 and 2012 from the demographic database, of which we were able to genetically sample 376 (96.9%). Paternity was assigned through a combination of exclusion and likelihood methods (Widdig et al. 2017). For 1602 of the 1649 infants (97.1%), the male assigned as the sire had no mismatch with the respective mother-infant dyad, while all other potential sires were excluded by two or more loci (“strict exclusion rule”). For a further 10 infants, the male assigned as the sire had no mismatch with the respective mother-infant dyad, while all other potential sires were excluded by one locus (“relaxed exclusion rule”). Finally, for the remaining 37 infants, the assigned sire had one mismatch with the respective mother-infant dyad; however, all other potential sires were excluded by at least two loci. Regardless of the rule used for paternity exclusion, for all 1649 infants, paternity was additionally supported at a 95% confidence level by the maximum likelihood method used by Cervus 3.0 (Kalinowski et al. 2007). Although it was not possible to apply blinded methods for the collection of genetic samples, genetic analysis was performed by individuals uninformed about the research question.

### Male group membership

When male dispersal was identified, the new group of residence was assigned if it remained constant for at least 30 days after the male was first observed in the new group. Only if males were observed to prospect other social groups, visit bachelor groups, or if they remained solitary (E. Dávila, personal observations) for some time within these first 30 days, the male’s residence status remained under close observation and was only assigned to a new social group once it remained constant for at least 60 days. Once residence was stable, the first day the animal was seen in the new group was defined as the date of immigration. Males were assigned to only one group on a given day. We then determined male group membership (in days) for all individuals present on the island at the time of conception of a given infant. It was not possible to record demographic data blind for determining group residency because our study involved animals in the field.

### Identifying extra-group paternity

To accurately identify EGPs, we defined whether sires were a resident or not in a group at the time of offspring conception. For this purpose we determined the estimated time of conception (hereafter conception window) for each offspring born in our study period by subtracting 166.5 ± 7.4 days (mean ± SD) from its date of birth (gestation length provided by Silk et al. 1993). Hence, for each offspring we obtained a conception window of 15 days.

To avoid assigning an EGP to a possible migratory event, we included not only the conception window but also 30 days prior to and 30 days after the conception window (i.e., a total of 75 days) into our definition of EGP. An offspring was considered to be the result of an EGP if it was sired by a male that was not a member in the offspring’s group according to the above definition during any of these 75 days. Accordingly, an offspring sired by a male that was a member of the offspring’s group throughout the 75 days or by a male that emigrated from or immigrated into the offspring’s group any time during the 75 days was considered to be a within-group offspring.

## Data analysis

To determine the causes of variation in the occurrence of EGPs, we determined the following variables for each social group and birth cohort:

- *Breeding group size*. To determine group size, we considered all sexually mature males and females present in a given group that were older than 1250 days (based on earliest age at reproduction: Bercovitch et al. 2003) at the start of the respective mating season.
- *Breeding group sex ratio*. To determine the sex ratio per breeding group, we counted the number of sexually mature adult females and males per group and mating season. We scored the sex ratio as the average number of adult females divided by the average number of adult males.
- *Female Synchrony*. To estimate whether the occurrence of EGP varied as a function of female synchrony (as estimated from live births only), we first determined for a particular group and season the number of unique dates across the entire mating season on which at least one female was estimated to be in estrous based on conception windows (determined by subtracting gestation duration from birth dates as described above). For example, if two females were present and the last 2 days of the first female’s 15-day estrous period coincided with the first 2 days of the second female’s estrous period, the number of unique estrous dates would be 28. We then divided 15 by this number, which essentially creates a proportion of all unique days that fit into a single 15-day conception window. Thus, if all females shared the exact same conception window, they are completely synchronous and the measure would produce a value of 15 days in one conception window/15 unique estrous days = 1. If, for example, there were five females in a group and there was no synchrony (i.e., no overlap of days at all), the value would decrease to 15 days in one conception window/75 unique estrous days = 0.2. Since miscarriages were not systematically observed and, hence, not included in this analysis, we could only consider females that were observed with a live birth, which ultimately provided us with a proxy of synchrony per group and season. We consider our proxy to accurately represent female synchrony given that on average, 86% of females give birth every year in the study population (Ruiz-Lambides, unpublished data).
- *Group instability*. We accounted for short-term fluctuations in a group by taking into account the deaths of males and males that emigrated (i.e., “membership loss”) as well as male immigrants (i.e., “membership gain”). We accounted not only for the presence or absence of males but also for how long they had been a member of a group. The duration of male group membership is highly predictive of rank (Vessey 1984; Berard 1999) because male rhesus macaques queue for dominance rather than achieve a higher position by means of fights (Vessey 1984; Dubuc et al. 2012).

In particular, for each group and season, a measure of membership loss was calculated by first identifying the males that were present on each day and the males that were present on the previous day. Males that were present on the previous day but not the current day represent the membership loss for the current day, measured as the sum of those missing males’ group membership durations. We then divided this membership loss value by the sum of all males’ membership durations from the previous day, creating the proportion of membership loss for the current day. The reasoning behind this was that the loss of a recently immigrated male presumably creates less instability than loss of a male that had been a member of the group for many years. Finally, we calculated the weighted average of these proportions across all days in the mating season, where the proportions were weighted by the inverse time lag to the onset of the mating season, as we regarded destabilization events to have a higher impact on the group when they were closer to the onset of the mating season.

Similarly, to obtain a measure of membership gain for each group and season, for every day in the mating season, we identified the males that were present on the current day but absent on the previous day. This number of new males was then divided by the total group size for the current day, resulting in a proportion of the group size that had joined the group that day. We then determined the average proportion across all days in the mating season, again weighted by the inverse time lag to the start of the mating season. The final measure of instability was a sum of the two weighted averages of membership loss and gain.

### Home range overlap

Home ranges of the six groups of our study population overlapped to varying degrees, providing different opportunities for interactions with other groups which also depend on the size of the other groups. To create a measure representing this overlap per season and group and also accounting for the size of the overlapping groups we proceeded as follows: First, we defined each group’s home range as the minimum convex polygon based on daily records by census takers (Online Resource 1). Second, we then laid a grid over the home range of a given group and determined the male density per grid cell of a home range as the number of males in the group, divided by the number of grid cells the home range comprised. As a measure of overlap of a given group’s home range with that of the other groups, we then determined for each grid cell in the given group’s home range the male density of the other groups and finally summed these densities over the entire home range. Hence, our measure of home range overlap was the summed male density of other groups in a given group’s home range.

### Statistical analyses

To test the group-level variables that may have influenced the occurrence of extra-group paternity, we applied a Generalized Linear Mixed Model (GLMM) (Baayen 2008) with Poisson error structure and log link function. The response variable was the number of offspring in a given group and season that was the result of extra-group paternity, resulting in 54 data points (nine birth cohorts x six social groups). As the number of extra-group offspring trivially increases with the total number of offspring born per group and season as well as the degrees of home range overlap, we controlled for both variables by including them (log transformed) into the model as offset terms (McCullagh and Nelder 1989). We explicitly decided to use home range overlap as an offset rather than as a predictor since the geographical constraints of this island population may translate into greater home range overlap, and consequently, this may have a trivial effect on rates of EGP in this population. Hence, our model investigates which factors affected rates of EGP when home range overlap is controlled for but does not estimate a separate coefficient for this predictor.

**Fig. 1:**
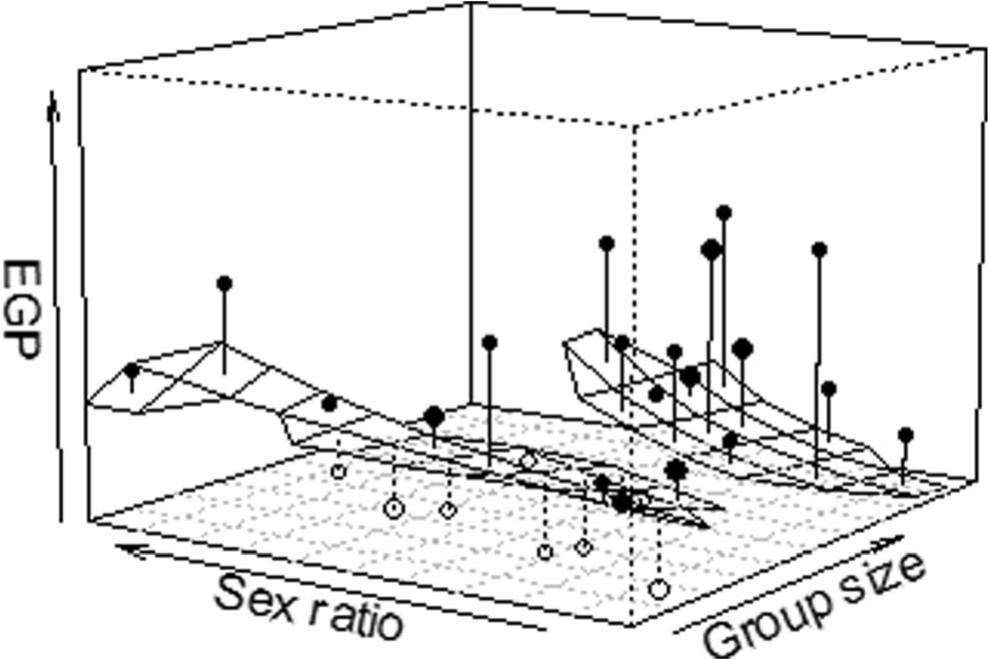
Interaction between breeding group sex ratio and group size. The plane illustrates the modeled influence for areas supported by data points; *points* show the response, averaged per model grid, with *filled points* depicting average responses above the model plane, and *open points* depicting a response below the model plane. The volume of the points represents sample size

We included the following predictor variables as fixed effects in order to model group-level characteristics potentially influencing the degree of EGPs: (i) group size, (ii) breeding group sex ratio, (iii) female synchrony, and (iv) group instability. As both the number of females and males and their ratios are important for our question, we also included the two-way interaction between sex ratio and group size. Notably, however, our data set did not comprise large groups with a pronounced female bias in the sex ratio (see Fig. 1) which could potentially compromise the technical soundness of fitting the interaction. As we regarded the interaction to be highly biologically meaningful, we fitted the interaction nonetheless, but also fitted a reduced model identical to our main model except that the interaction between sex ratio and group size was omitted. The aim of the reduced model was to evaluate the effects of sex ratio and group size when not comprised in the interaction since the results for these predictors obtained from the model including the interaction might not be trustworthy. All the predictor variables were covariates that were standardized to a mean of zero and a standard deviation of one (Schielzeth 2010). In addition, we included group ID and birth cohort as random effects in the model. To keep type I error rates at 5%, we included random slopes for each main effect within each random effect (Barr et al. 2013). Our model estimated 15 parameters in total, including random intercepts, random slopes, and offset terms. Due to the recent debate about fitting maximal random slope structures, we further repeated the model without random slopes (Online Resource 2 Table S1). Results of the model with and without random slopes showed similar estimates and suggested the same patterns; we therefore present the details of the model including random slopes in the main text and results of the model without random slopes in the online resource only.

The GLMM was fitted using the function “glmer” provided by the package “lme4” (Bates et al. 2014) in R 3.0.2 (R Development Core Team 2013). The model including the interaction appeared to be slightly overdispersed (dispersion parameter 1.202) while the reduced model excluding the interaction was slightly underdispersed (dispersion parameter 0.747). Collinearity was not an issue as indicated by Variance Inflation Factors (VIF) (Quinn and Keough 2002). VIFs were derived from a model lacking the random effects and the interaction (largest VIF = 2.3) and were determined using the function “vif” of the R package “car”; (Fox and Weisberg 2011). We assessed model stability by excluding each group and cohort from the data one at a time and fitting the model for each of the derived data sets. The estimates obtained from these data sets were similar to those obtained for the full data set, indicating that the model was stable.

Applying Likelihood Ratio Tests (hereafter LRT) (Dobson 2002) (R function “anova”), we first determined the statistical significance of the full model by comparing its fit to that of the null model (comprising only the random effects, random slopes, and offset terms). We also assessed the significance of the predictor variables, including the interaction, using LRTs (R function “drop1”). We considered *P* values <0.05 as significant and 0.05 < *P* < 0.1 as a trend. Trends should not be overlooked, since dichotomizing results based on *P* values being significant or not can result in misleading conclusions (Stoehr 1999).

## Results

Out of the 1649 offspring considered in the study, we were able to identify a total of 264 (16%) extra-group paternities using the criteria defined above. Rates of EGP per group and season ranged from 0 to 64.7% (see Table 1), with mean group size ranging from 29 to 166 adult individuals and the breeding group sex ratio from 1.1 to 2.7 (i.e., female-biased) (for detailed socio-demographic information per group and season see Online Resource 2 Table S2).

**Table 1:** Percentages of extra-group paternities among six groups of rhesus macaques studied between 2004 and 2012. *N* represents the number of EGPs/number of within-group offspring, % gives the percentage of EGPs out of all genotyped offspring in a given group and season. Groups are ordered from highest to lowest average group size throughout the years

Year	Group													
	F		R		KK		HH		V		S		Total	
	*N*	%	*N*	%	*N*	%	*N*	%	*N*	%	*N*	%	*N*	%
2004	4/47	7.8	7/43	14	6/11	35.3	3/6	33.3	1/15	6.3	3/10	23.1	24/132	15.4
2005	11/57	16.2	2/53	3.6	7/12	36.8	0/10	0.0	0/13	0.0	8/6	57.1	28/151	15.6
2006	5/52	8.8	7/54	11.5	6/15	28.6	4/9	30.8	1/11	8.3	2/6	25.0	25/147	14.5
2007	1/55	1.8	12/29	29.3	4/18	18.2	9/8	52.9	0/15	0.0	2/15	11.8	28/140	16.7
2008	4/52	7.1	21/30	41.2	1/21	4.5	5/19	20.8	0/15	0.0	4/5	44.4	35/142	19.8
2009	4/58	6.5	4/54	6.9	7/18	28.0	0/22	0.0	0/22	0.0	8/12	40.0	23/186	11.0
2010	12/47	20.3	3/53	5.4	7/20	25.9	7/24	22.6	0/23	0.0	10/11	47.6	39/178	18.0
2011	4/46	8.0	3/32	8.6	2/23	8.0	3/30	9.1	1/22	4.3	11/6	64.7	24/159	13.1
2012	13/40	24.5	11/42	20.8	6/14	30.0	1/17	5.6	0/27	0.0	7/10	41.2	38/150	20.2
Total	58/454	11.3	70/390	15.2	46/152	23.2	32/145	18.1	3/163	1.8	55/81	40.4	264/1385	16.0

Overall, the set of predictor variables tended to influence the number of EGPs (LRT, χ^2^ = 9.44, df = 5, *P* = 0.093). The results suggested an interaction between sex ratio and group size. Specifically, the EGP rate was particularly low in large groups when the sex ratio was low (male-biased) and tended to increase as the proportion of females increased. In small groups, on the other hand, the EGP rate was moderately high throughout and seemed to be barely influenced by sex ratio (Table 2, Fig. 1). With the respective other predictor being at its average, the estimates for sex ratio and group size showed the same pattern (i.e., a positive effect of sex ratio and a negative effect of group size on the number of EGPs) irrespective of whether their interaction was included into the model or not (Table 2). When the interaction was excluded from the model, the effects of both predictors were non-significant, indicating that sex ratio and group size independently did not have obvious effects on rates of EGP but that they indeed might have interacted.

**Table 2:** Results of the GLMM of social group effects on numbers of EGPs (main model, unless denoted otherwise)

Predictor variable	Estimate	Standard error of estimate	*χ* ^2^	Degrees of freedom	*P* value
Intercept	3.21	0.17			
Sex ratio	0.39	0.11			
Sex ratio (reduced model)	0.20	0.16	1.41	1.00	0.235
Group size	−0.36	0.30			
Group size (reduced model)	−0.29	0.19	1.10	1.00	0.295
Sex ratio × group size	0.31	0.11	3.00	1.00	0.083
Female synchrony	−0.37	0.17	4.59	1.00	0.032
Group instability	−0.26	0.11	3.64	1.00	0.056

Mating season and the social group were included as random effects. All test predictors (i.e., breeding group size, breeding group sex ratio, female synchrony, group instability) were included as random slopes within mating season and social group and were *z*-transformed to a mean of 0 and a standard deviation of 1; mean ± SD of the original variables were 1.615 ± 0.318 (sex ratio), 82.157 ± 43.683 (group size), 0.140 ± 0.039 (female synchrony), and 0.00037 ± 0.00031 (group instability). LRT results not shown for intercept and variables included in an interaction because these have a very limited interpretation

**Fig. 2:**
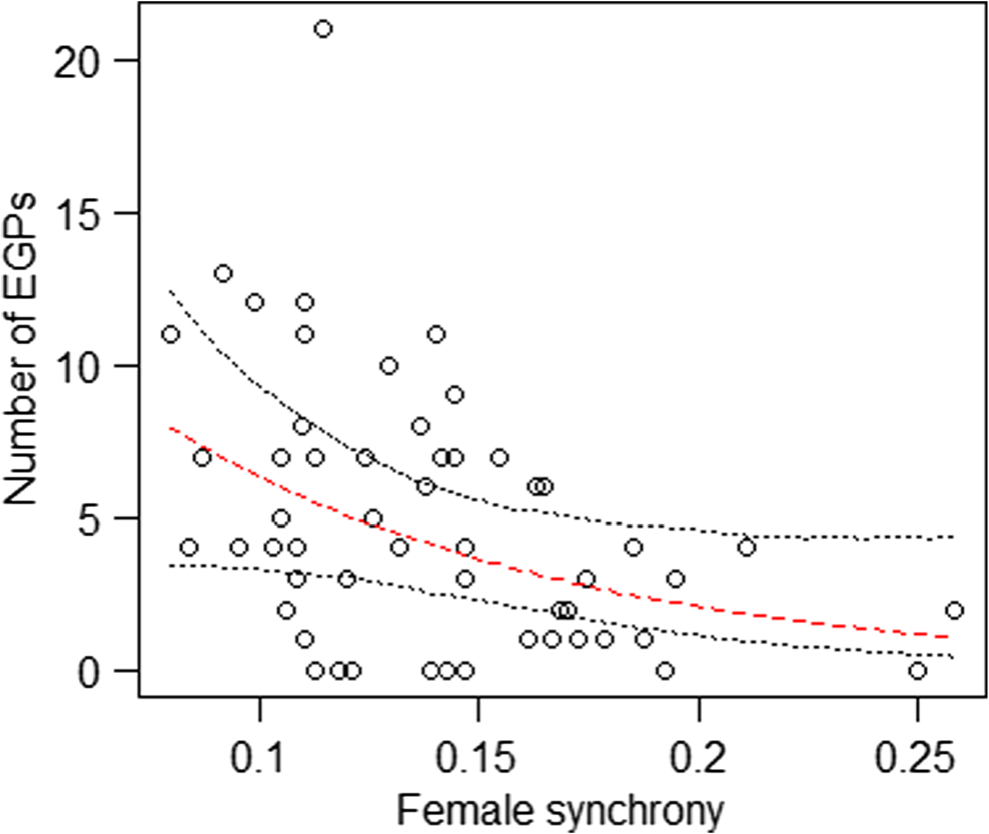
Impact of female reproductive synchrony on the number of EGPs. *Points* show the raw data, the lines indicate the fitted model (*dashed*, *red*) and its confidence limits (*dotted*, *black*)

**Fig. 3:**
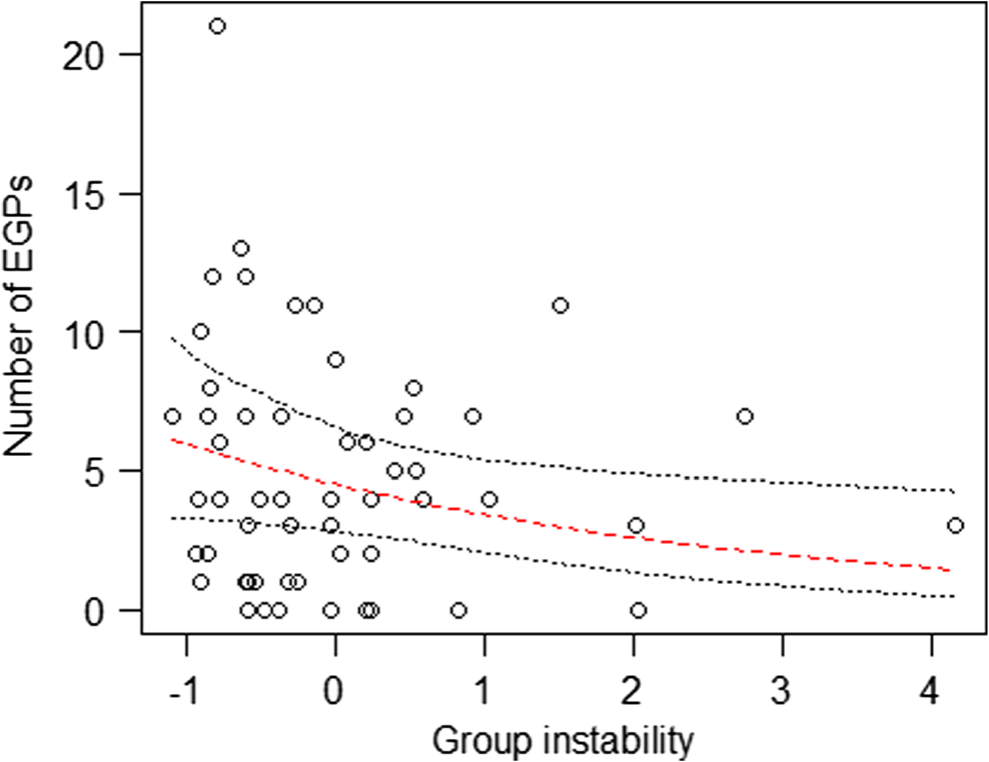
Impact of group instability (*z*-transformed) on the number of EGPs in social groups. *Points* show the raw data, the *lines* indicate the fitted model (*dashed, red*) and its confidence limits (*dotted*, *black*)

There seemed to be fewer EGPs as female synchrony increased (Table 2, Fig. 2). Finally, EGPs tended to be less common in social groups that were more unstable in their group composition (Table 2, Fig. 3).

## Discussion

Our longitudinal analysis of the causes of EGPs in a nonhuman primate population revealed high variation in the number of EGPs across groups and years, and as predicted, the variation tended to depend on particular aspects related to group structure. Our results expand previous findings and highlight the fact that EGPs can potentially constitute a substantial part of annual male reproduction. Therefore, EGPs possibly affect the distribution of paternity within social groups and, thus, should be taken into account in studies of male reproductive success in group-living mammals.

Interestingly, in the present analysis, results point towards a complex interplay between the effects of group size and sex ratio on the rate of EGPs. Although our data set did not cover large groups with a pronounced female bias, results suggest that EGPs tended to be more frequent when the sex ratio was relatively even rather than male-biased when group size was large. In small groups, on the other hand, EGPs did not appear to be affected much by whether the sex ratio was male-biased or female-biased. This suggests that in large groups, within-group males may not be able to closely monitor all females if their numbers equal or slightly surpass those of the males, which may increase a female’s possibilities to sneak away and engage in extra-group mating (Clutton-Brock and Isvaran 2007). This possible interaction between sex ratio and group size might be one explanation why some earlier studies investigating patterns of EGP across different species found different results with regard to sex ratio and group size than we did in our study, e.g., a weak relation between sex ratio and EGP (Isvaran and Clutton-Brock 2007) or no relation between group size and EGP (van Noordwijk and van Schaik 2004). Another possible explanation is that the interspecific patterns described in these earlier studies may differ from intraspecific dynamics of EGP. We further need to consider that our data set did not comprise large groups with a pronounced female-biased sex ratio which might deviate from the patterns we observed here. Overall, our results point to the possibility that sex ratio and group size do not independently affect the occurrence of EGP, but that they interact in doing so. Whether this result is extendable even to large groups with considerably more females than males needs to be investigated in future studies.

We had further predicted that, as the synchrony of estrous females increases, the number of EGPs would increase too, as we assumed that females would be more free to exhibit mate choice if males were engaged in mate-guarding other females. However, contrary to our prediction, the analysis suggested that the more synchronous births were in a given group and season, the fewer offspring resulted from EGPs. One potential explanation may be that greater synchrony in breeding females may reflect a shortened effective breeding season, which limits the period that males from other groups have to seek EGPs in that particular group (Westneat et al. 1990). At the same time, many simultaneously receptive females within a group may increase mating opportunities of lower-ranking within-group males when higher-ranking males are engaged in mate-guarding certain females. Mating with lower-ranking group males may be more achievable or less costly for females than seeking sexual partners outside their social group which could additionally account for the observed inverse relationship between female synchrony and numbers of EGP. Finally, we cannot fully exclude that the observed results are related to our measure of female synchrony being based on observed births rather than measures of actual estrous. However, given that in the studied population 86% of the females per year give birth, we expect our measure to also closely reflect estrous synchrony and, therefore, consider it highly unlikely that the observed patterns were driven by the way we measured synchrony. Yet, to better understand the relationship between female synchrony and EGPs, future studies should include female hormone data to measure female synchrony more precisely, and should investigate individual attributes (e.g., age, rank) of both males and females engaging in EGPs in addition to the group parameters considered in the present study.

We predicted that group instability would influence the number of extra-group paternities in a social group, such that levels of EGP increase as groups become more unstable when resident males emigrate or new males immigrate. Contrary to our predictions, however, the number of EGPs tended to increase the more stable the group composition was. If groups remain stable for extended periods of time, group members will become increasingly familiar with each other and may have mated with each other already in previous seasons. Accordingly, female rhesus macaques living in a stable group might pursue EGPs as an alternative reproductive strategy, i.e., avoiding males they have shared long co-residence and instead mating with novel males (Manson 1995) in order to enhance genetic variability for their offspring. For males, on the other hand, only major disturbances to the group may compromise their ability to monitor the females of their group and thus affect rates of EGP. For example, when the male hierarchy was unstable following the disruption of the queuing system through an alpha overthrow with significant increase of intragroup aggression, females may have become more accessible to non-group males, which in turn could explain the high rates of EGP observed in that particular group and year of our study population (59.3% EGPs: Georgiev et al. 2016). Future studies will be needed to validate the results obtained in the present study and to investigate whether measures of hierarchy stability might give additional clues to our measure of group instability that addressed changes in group composition but not changes in rank of the group members.

Finally, we included group overlap as an offset, based on the assumption that a large home range overlap may increase rates of EGP. Indeed we noticed a difference in EGP rates in two small groups (compare Table 1, groups S and V) that barely differed in size throughout birth seasons 2004–2012, but the extent of home range overlap of group S in comparison with group V was considerably higher. Being included as an offset, our model tested the effects of group composition on EGP independent from group overlap and we encourage future studies to also incorporate home range overlap into their analyses.

In conclusion, our study suggested variation in EGP being influenced by group structure, i.e., group size, breeding group sex ratio, female synchrony, and group instability, which may be interpreted as a consequence of individual reproductive strategies (Clutton-Brock 2009). In particular, the patterns described in our study suggest that numbers of EGP may be affected by male mating opportunities and/or limited male monopolization potential but also by the possibility for females to pursue EGPs as an alternative reproductive strategy to possibly seek more genetically compatible males to ultimately outbreed (Bateson 1983). As such, these results might be suggestive of female mate choice as proposed in earlier work on rhesus macaques (Chapais 1983; Manson 1992). More studies are needed to verify the suggested patterns and should address the male and female perspective separately to further elucidate whether males or females (or both) preferentially search for EGPs.

## Electronic supplementary material

ESM 1 (PDF 534 kb)

ESM 2 (DOCX 22 kb)
