# Supplementary material for: Long-term analysis on the variance of extra-group paternities in rhesus macaques
Source: Behav Ecol Sociobiol. 2017 Mar 17;71(4):67. doi: 10.1007/s00265-017-2291-7 (PMC5355504; doi:10.1007/s00265-017-2291-7)
Supplement: Supplementary file 1 — (PDF 534 kb) [file 265_2017_2291_MOESM1_ESM.pdf]

Electronic Supplementary Material 1

Long-term analysis on the variance of extra-group paternities in rhesus macaques

Behavioral Ecology and Sociobiology

Angelina V. Ruiz-Lambides[1,2,3], Brigitte M. Weiß[1,2], Lars Kulik[1,2], Colleen Stephens[4], Roger Mundry[4], Anja Widdig[1,2]

* Corresponding author: Angelina V. Ruiz-Lambides, Junior Research Group of Primate Kin Selection, Department of Primatology, Max-Planck Institute for Evolutionary Anthropology, Behavioral Ecology Research Group, Institute of Biology, Faculty of Bioscience, Pharmacy and Psychology, University of Leipzig, Cayo Santiago Field Station, Caribbean Primate Research Center, University of Puerto Rico. Email: angelina.ruiz@upr.edu

Each of the 6 social group’s home range depicted as the minimum convex polygon based on daily records by census takers

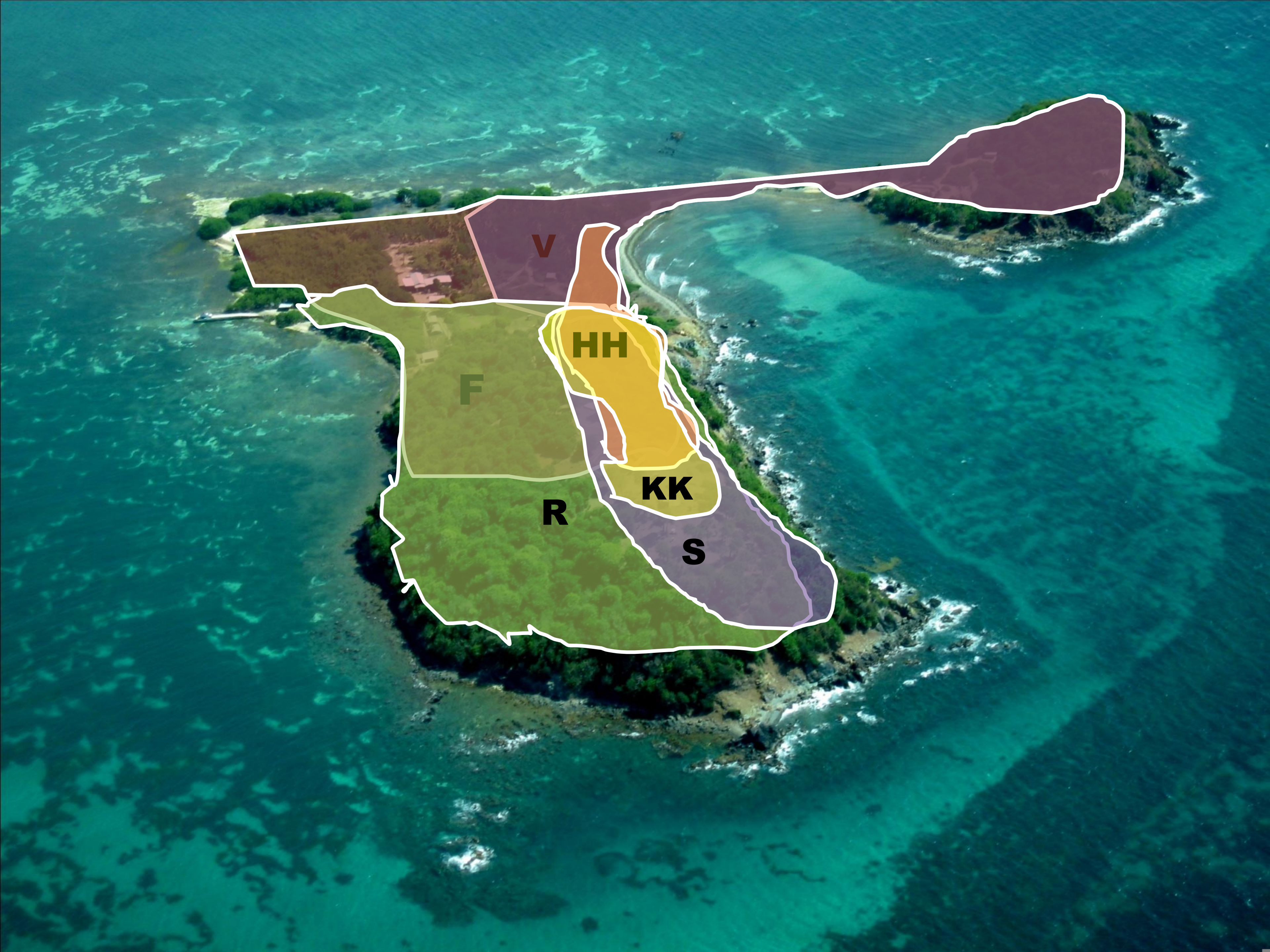
V
HH
F
KK
R
S
