# Supplementary material for: Long-term analysis on the variance of extra-group paternities in rhesus macaques
Source: Behav Ecol Sociobiol. 2017 Mar 17;71(4):67. doi: 10.1007/s00265-017-2291-7 (PMC5355504; doi:10.1007/s00265-017-2291-7)
Supplement: Supplementary file 2 — (DOCX 22 kb) [file 265_2017_2291_MOESM2_ESM.docx]

**Electronic Supplementary Material 2**

**Long-term analysis on the variance of extra-group paternities in rhesus macaques**

Behavioral Ecology and Sociobiology

Angelina V. Ruiz-Lambides^1,2,3^, Brigitte M. Weiß^1,2^, Lars Kulik^1,2^, Colleen Stephens^4^, Roger Mundry^4^, Anja Widdig^1,2^

* Corresponding author: Angelina V. Ruiz-Lambides, Junior Research Group of Primate Kin Selection, Department of Primatology, Max-Planck Institute for Evolutionary Anthropology, Behavioral Ecology Research Group, Institute of Biology, Faculty of Bioscience, Pharmacy and Psychology, University of Leipzig, Cayo Santiago Field Station, Caribbean Primate Research Center, University of Puerto Rico. Email: angelina.ruiz@upr.edu

**Table S1** Results of the GLMM of social group effects on numbers of EGPs (without random slopes).* Mating season and the social group were included as random effects. LRT results not shown for intercept and variables included in an interaction because these have a very limited interpretation. The full null model comparison revealed χ ^2^=22.829, df=5, p<0.001.

| Predictor variable | Estimate | Standard error of estimate | χ ^2^ | Degrees of freedom | *P value* |
| --- | --- | --- | --- | --- | --- |
| Intercept | 3.55 | 0.23 |  |  |  |
| Sex Ratio | 0.32 | 0.11 |  |  |  |
| Group Size | -0.39 | 0.19 |  |  |  |
| Sex ratio x group size | 0.22 | 0.11 | 3.86 | 1.00 | 0.049 |
| Female Synchrony | -0.32 | 0.13 | 6.86 | 1.00 | 0.008 |
| Group Instability | -0.15 | 0.08 | 3.07 | 1.00 | 0.079 |

*All predictors were z-transformed to a mean of 0 and a standard deviation of 1; mean + SD of the original variables were 1.615 + 0.318 (sex ratio), 82.157 + 43.683 (group size), 0.140 + 0.039 (female synchrony), and 0.00037 + 0.00031 (group instability)

**Table S2** Socio-demographic group parameters across birth seasons 2004 – 2012

| Year | Group | Breeding group sex ratio | Mean Group size | Group instability | Female synchrony |
| --- | --- | --- | --- | --- | --- |
| 2004 | F | 1.9 | 137.1 | 6.91E-01 | 0.096 |
| 2004 | HH | 1.2 | 30.8 | 9.95E-04 | 0.174 |
| 2004 | KK | 1.9 | 38.3 | 3.91E-04 | 0.138 |
| 2004 | R | 1.8 | 120.1 | 6.52E-04 | 0.105 |
| 2004 | S | 1.1 | 42.5 | 2.70E-04 | 0.195 |
| 2004 | V | 1.5 | 46.1 | 2.65E-04 | 0.172 |
| 2005 | F | 2 | 134.2 | 8.40E-04 | 0.080 |
| 2005 | HH | 1.9 | 29 | 4.37E-04 | 0.250 |
| 2005 | KK | 2.7 | 39 | 1.23E-03 | 0.144 |
| 2005 | R | 1.6 | 125.7 | 9.80E-05 | 0.106 |
| 2005 | S | 1.1 | 36.1 | 5.31E-04 | 0.110 |
| 2005 | V | 1.3 | 45.4 | 2.47E-04 | 0.192 |
| 2006 | F | 1.8 | 143.9 | 5.32E-04 | 0.105 |
| 2006 | HH | 1.6 | 35.5 | 5.51E-04 | 0.147 |
| 2006 | KK | 2.1 | 47.5 | 4.31E-04 | 0.163 |
| 2006 | R | 1.7 | 122.3 | 9.50E-05 | 0.087 |
| 2006 | S | 1.7 | 35.9 | 3.76E-04 | 0.259 |
| 2006 | V | 1.2 | 47.6 | 1.82E-04 | 0.161 |
| 2007 | F | 1.7 | 148.7 | 1.76E-04 | 0.110 |
| 2007 | HH | 1.4 | 45.8 | 3.67E-04 | 0.144 |
| 2007 | KK | 1.8 | 51.6 | 1.19E-04 | 0.185 |
| 2007 | R | 1.4 | 126.3 | 1.76E-04 | 0.110 |
| 2007 | S | 1.8 | 34.3 | 7.20E-05 | 0.169 |
| 2007 | V | 1.2 | 48.3 | 1.78E-04 | 0.118 |
| 2008 | F | 1.8 | 143.9 | 4.40E-04 | 0.109 |
| 2008 | HH | 1.4 | 60.7 | 4.90E-04 | 0.126 |
| 2008 | KK | 1.6 | 63.1 | 7.90E-05 | 0.167 |
| 2008 | R | 1.7 | 136.4 | 1.13E-04 | 0.115 |
| 2008 | S | 2 | 38.7 | 7.40E-05 | 0.211 |
| 2008 | V | 1.2 | 55.5 | 4.29E-04 | 0.113 |
| 2009 | F | 1.8 | 152.1 | 2.48E-04 | 0.084 |
| 2009 | HH | 1.4 | 72.7 | 6.22E-04 | 0.121 |
| 2009 | KK | 1.4 | 63 | 2.30E-05 | 0.142 |
| 2009 | R | 1.5 | 152.9 | 2.04E-04 | 0.103 |
| 2009 | S | 1.9 | 47.7 | 1.02E-04 | 0.136 |
| 2009 | V | 1.5 | 60.3 | 1.00E-03 | 0.139 |
| 2010 | F | 1.8 | 166.6 | 1.07E-04 | 0.099 |
| 2010 | HH | 1.3 | 81.1 | 1.74E-04 | 0.113 |
| 2010 | KK | 1.6 | 66.2 | 5.07E-04 | 0.124 |
| 2010 | R | 1.2 | 152 | 1.82E-04 | 0.120 |
| 2010 | S | 1.7 | 52.6 | 8.00E-05 | 0.129 |
| 2010 | V | 1.4 | 67.5 | 3.56E-04 | 0.147 |
| 2011 | F | 1.7 | 149.4 | 3.54E-04 | 0.132 |
| 2011 | HH | 1.5 | 81.7 | 1.67E-03 | 0.109 |
| 2011 | KK | 1.4 | 76.3 | 4.40E-04 | 0.171 |
| 2011 | R | 1.5 | 122.5 | 3.54E-04 | 0.147 |
| 2011 | S | 2.5 | 43.5 | 2.79E-04 | 0.140 |
| 2011 | V | 1.5 | 60.6 | 1.96E-04 | 0.179 |
| 2012 | F | 1.7 | 150.1 | 1.67E-04 | 0.092 |
| 2012 | HH | 1.5 | 83.2 | 2.87E-04 | 0.188 |
| 2012 | KK | 1.4 | 77.3 | 1.18E-04 | 0.165 |
| 2012 | R | 1.7 | 131.7 | 3.19E-04 | 0.110 |
| 2012 | S | 1.9 | 45.7 | 2.51E-04 | 0.155 |
| 2012 | V | 1.3 | 69.5 | 2.14E-04 | 0.143 |
